# Exploring predictors of depressive symptoms in patients with multiple sclerosis: The effect of neuropathic pain, shame, and mindfulness

**DOI:** 10.1192/j.eurpsy.2021.307

**Published:** 2021-08-13

**Authors:** T. Carvalho, L. Benedito, C. Gomes

**Affiliations:** 1 Psychology, Instituto Superior Miguel Torga, Coimbra, Portugal; 2 Faculty Of Psychology And Educational Sciences, University Of Coimbra, Center for Research in Neuropsychology and Cognitive-Behavioral Intervention (CINEICC), Coimbra, Portugal; 3 Clínica de Saúde Psiquiátrica de Coimbra – Casa da Oliveira, Coimbra, Portugal

**Keywords:** Multiple sclerosis, depressive symptoms, predictive model

## Abstract

**Introduction:**

Multiple Sclerosis (MS) is a chronic inflammatory, immune-mediated, demyelinating disease of the central nervous system, with a progressive course. It is potentially disabling and affects mainly young adults. Depression is the mental disorder with the greatest comorbidity with MS and tends to worsen its symptomatology and course. However, knowledge about the predictors of depression in patients with MS is scarce.

**Objectives:**

This preliminary study aimed to verify whether neuropathic pain (NP), internal (IS) and external (ES) shame and mindfulness predict depressive symptoms in patients with MS.

**Methods:**

This cross-sectional study included a convenience sample of 95 patients diagnosed with MS and without other identified neurological diseases. Participants completed the Depression Subscale of the Depression, Anxiety and Stress Scales-21, the Analogue Pain Scale of the Pain Detect Questionnaire, the External and Internal Shame Scale, and the Mindfulness Subscale of the Self-Compassion Scale.

**Results:**

All potential predictors exhibited significant correlations with depressive symptoms and significantly predicted this symptomatology in simple linear regression models. Thus, they were included as covariates in the multiple linear regression model. This model explained a high percentage of the variance of depressive symptoms (40.5%) and identified NP, IS and mindfulness as significant predictors.

**Conclusions:**

Interventions aimed at preventing/reducing depression in patients with MS should minimize IS and develop mindfulness and NP coping skills, in order to promote mental health in this target population and possibly prevent the exacerbation and progression of MS symptomatology.

**Disclosure:**

No significant relationships.

